# Total laparoscopic and thoracoscopic Ivor Lewis esophagectomy after neoadjuvant Chemoradiation with minimal overall and anastomotic complications

**DOI:** 10.1186/s13019-019-0937-4

**Published:** 2019-06-28

**Authors:** Robert E. Merritt, Peter J. Kneuertz, Desmond M. D’Souza, Kyle A. Perry

**Affiliations:** 1 Division of Thoracic Surgery, The Ohio State University Wexner Medical Center, N847 Doan Hall, 410 West 10th Avenue, Columbus, OH 43210 USA; 2 Division of General and Gastrointestinal Surgery, The Ohio State University Wexner Medical Center, N847 Doan Hall, 410 West 10th Avenue, Columbus, OH 43210 USA

**Keywords:** Minimally invasive Esophagectomy, Esophageal Cancer, Induction therapy

## Abstract

**Background:**

The published rates of morbidity and mortality remain relatively high for patients who undergo laparoscopic and thoracoscopic Ivor Lewis esophagectomy. We report the postoperative and oncologic outcomes of a large cohort of patients with esophageal carcinoma who were uniformly treated with laparoscopic and thoracoscopic Ivor Lewis esophagectomy following neoadjuvant chemoradiation.

**Methods:**

This is a retrospective observational study of 112 patients diagnosed with esophageal carcinoma who underwent total laparoscopic and thoracoscopic Ivor Lewis esophagectomy from May 2014 to May 2018. All of the patients received neoadjuvant chemoradiation consisting of 45 to 50.4 Gray of radiation and 3–5 cycles of carboplatin and paclitaxel chemotherapy. Perioperative morbidity and 90-day mortality were recorded. The overall and disease-free survival rates were estimated by Kaplan Meier techniques.

**Results:**

A total of 112 patients completed induction chemoradiation followed by a total laparoscopic and thoracoscopic Ivor Lewis esophagectomy. There were 87 (77.68%) males and 25 (22.32%) females with a mean age of 61.6 years ± 10.4. A total of 28 (25%) patients had one or more complications. A total of 4 patients (3.57%) had an anastomotic leak. The 90-day mortality rate was 0.89%. The 3-year overall survival rate was 64.7% and the 3-year disease-free survival rate was 70.2%.

**Conclusion:**

The current outcomes suggest that laparoscopic and thoracoscopic Ivor Lewis esophagectomy can be performed with minimal overall and anastomotic complications following neoadjuvant chemoradiation.

## Background

In 2016, there were approximately 16,910 projected new cases of esophageal carcinoma and 15,910 projected associated deaths [1]. The majority of resectable esophageal carcinoma cases present with locally advanced disease. The randomized Chemoradiotherapy for Oesophageal Cancer followed by Surgery Study (CROSS) Trial demonstrated improved overall survival and disease-free survival in patients who underwent preoperative chemoradiotherapy follow by esophagectomy [2]. The most common operative approach for esophageal carcinoma of the distal third of the esophagus and the gastroesophageal junction is the Ivor Lewis esophagectomy, which involves a laparotomy, right thoracotomy, and an intra-thoracic anastomosis [3, 4]. In recent years, the total laparoscopic and thoracoscopic Ivor Lewis esophagectomy has become more prevalent and has been shown to result in fewer respiratory complications [5–8]. Despite the decreased respiratory complications, the incidence of anastomotic complications remain moderately high even with minimally invasive esophagectomy [9]. Anastomotic leaks result in significant morbidity and mortality and directly cause increased hospital length of stay. Anastomotic and other complications have been shown to negatively influence overall survival for esophageal carcinoma after esophagectomy [10].

Some reports have demonstrated evidence that preoperative chemoradiation may increase the incidence of anastomotic complications after esophagectomy [11, 12]. This study describes the results of a large cohort of patients who underwent neoadjuvant chemoradiation followed by total laparoscopic and thoracoscopic Ivor Lewis esophagectomy for locally advanced esophageal carcinoma. In this study, all of the patients underwent laparoscopic gastric devascularization at least 2 weeks prior to esophagectomy. The gastric ischemic preconditioning may result in improved gastric conduit perfusion and should mitigate any deleterious effects of neoadjuvant chemoradiation on anastomotic healing [13]. The minimization of overall and anastomotic complications may further improve the overall and disease-free survival for esophageal carcinoma after neoadjuvant chemoradiation followed by total thoracoscopic and laparoscopic esophagectomy.

## Materials and methods

### Study design

This study is a retrospective observational study of 112 patients who underwent total thoracoscopic and laparoscopic Ivor Lewis esophagectomy for esophageal carcinoma at the Ohio State Wexner Medical Center between May 2014 and June 2018. During this period, 171 Ivor Lewis esophagectomy procedures were performed for esophageal carcinoma. There were 41 open (right posterior lateral thoracotomy) and 130 laparoscopic and thoracoscopic Ivor Lewis esophagectomies performed. We selected 112 patients who received neoadjuvant chemoradiation before undergoing a laparoscopic and thoracoscopic Ivor Lewis Esophagectomy for this study. The charts and electronic medical records of all patients were reviewed and data was collected in a de-identified fashion. The study was approved by the Ohio State Institutional Review Board and the requirement for informed consent were waived. All of the patients were clinically staged before the initiation of treatment with endoscopy, Computed Tomography (CT) scans, clinical history, and physical exam. Endoscopic Ultrasound (EUS) was performed in 83.93% (94/112) of the patients and Positron Emission Tomography (PET) scans were performed in all 112 (100%) patients prior to esophagectomy. All of the patients received neoadjuvant chemoradiation prior to total thoracoscopic and laparoscopic esophagectomy. The neoadjuvant chemoradiation regimen consisted of Carboplatin and Paclitaxel and concurrent radiation doses that ranged from 45 Gray to 50.4 Gray. The neoadjuvant chemoradiation regimen was completed in 6–8 weeks.

All of the patients underwent preoperative risk assessment with a cardiac stress test, pulmonary function test, and a history and physical. Cardiac comorbidity was recorded if there was a history of acute myocardial infarction or a previous coronary artery bypass grafting or percutaneous coronary stenting procedure. Chronic obstructive pulmonary disease (COPD) was recorded as a comorbidity if there was a prior diagnosis of asthma, emphysema, or chronic bronchitis. The preoperative ejection fractions were obtained from echocardiogram reports. The forced expiratory volume in 1 s (FEV1) and diffusion capacity (DLCO) percent predicted values were recorded from the preoperative pulmonary function test reports. Patients with an ECOG performance status score of greater than 2 were not typically selected to undergo laparoscopic and thoracoscopic Ivor Lewis esophagectomy. The patients underwent a diagnostic laparoscopy and gastric devascularization procedure a mean of 18.2 days ± 14.7 prior to esophagectomy. The left gastric artery, the coronary vein, and the short gastric vessels were divided during the gastric devascularization procedure [13].

Surgical technique for total laparoscopic and thoracoscopic Ivor Lewis esophagectomy.

The laparoscopic mobilization and preparation of the gastric conduit for all of the cases in this series was performed by author KAP, who is a minimal access general surgeon. The patient is positioned on the operating room table in the supine position. A double lumen tube, arterial line, and epidural catheter are placed by the anesthesiologist. An esophagogastroduodenoscopy (EGD) is performed to confirm the location of the esophageal tumor. Following needle insufflation of the abdomen, five laparoscopic ports are placed for the abdominal portion of the procedure. These include a 10 mm port to the left of the mid-line approximately 4 cm above the umbilicus, a 12 mm port in the left upper quadrant, a 5 mm port in the left mid-abdomen, and a 15-mm port in the right upper quadrant. A self-retaining retractor is placed in the epigastric position to retract the left lateral segment of the liver and expose the esophageal hiatus. The greater curvature of the stomach is mobilized by dividing the gastrocolic ligament using ultrasonic dissection while taking care to preserve the right gastroepiploic artery. This dissection is carried out to the level of the origin of the gastroepiploic artery. A formal Kocher maneuver is not performed unless required to allow the pylorus to reach the level of the esophageal hiatus. The gastrohepatic ligament is incised and the hiatus is dissected circumferentially and a Penrose drain is placed around the distal esophagus. The distal esophagus is mobilized to the level of the inferior pulmonary vein and care is taken to maintain the upper abdominal and lower mediastinal lymph nodes with the specimen. Following complete gastric mobilization, a 5 cm wide gastric conduit is created using multiple applications of the Endo GIA endoscopic stapler (Medtronic, Boulder, CO) along the lesser curvature. The gastric conduit is then sutured to the distal aspect of the esophagogastrectomy specimen. Gastric emptying procedures and feeding jejunostomy tube placement is not routinely performed.

The thoracoscopic portion of all of the cases were performed by author REM, who is a general thoracic surgeon. For the right thoracoscopy, the patients were positioned in the left lateral decubitus position and three right thoracoscopic ports and a small access incision without rib-spreading were utilized. A 12 mm port is placed in the 8th intercostal space posterior axillary line for the 10 mm thoracoscope. A 12 mm port is placed in the 5th intercostal space anterior-axillary line, and a 3 cm access incision is made in the 9th intercostal space for removal of the specimen and placement of the EEA circular stapler. A 12 mm port is placed below the tip of the scapula. The esophagus and the lymphatic tissue are dissected circumferentially from the hiatus to about 2 cm above the azygous vein. The esophagus is divided with a linear Endo GIA stapler at the level of the azygous vein. The conduit and specimen are then pulled gently into the right chest, taking great care not to twist the gastric conduit. A 25 mm anvil (OrVil, Medtronic, Boulder, CO) is passed trans-orally through a small esophagotomy in the esophageal stump staple line. The anastomosis is completed by joining the anvil with the 25 mm end-to-end anastomosis (EEA) stapler (Medtronic, Boulder, CO) inserted through a gastrotomy at the tip of the gastric conduit. The EEA stapler pin is deployed along the greater curvature and the esophagogastric anastomosis is created. A nasogastric tube is then passed under direct vision into the gastric conduit. The gastrotomy is then resected with 2–3 applications of the Endo GIA stapler. The anastomosis is covered with redundant omentum or mediastinal pleura. A barium swallow study is obtained on postoperative day number 6 to evaluate the esophagogastric anastomosis for a leak.

Postoperative complications were reported as anastomotic leak, conduit necrosis, anastomotic stricture, pneumonia, respiratory failure, pneumothorax, airway fistula, atrial fibrillation, chylothorax, and atelectasis. The Clavien-Dindo severity stratification system was used to describe the severity of postoperative complications [14]. Anastomotic leaks were diagnosed by observing extravasation of oral contrast at the esophagogastric anastomosis on a contrast esophagram and/or by direct clinical observation. Conduit necrosis was reported in cases involving complete anastomotic dehiscence and conduit ischemia requiring completion gastrectomy and esophageal diversion. Respiratory failure was defined as the need for re-intubation for isolated respiratory dysfunction during the postoperative period or the initiation of high flow oxygen for acute hypoxemia. Pneumonia was diagnosed if patients developed an infiltrate on chest imaging studies with associated fever and received antibiotic therapy. A postoperative mortality was defined as a death occurring during hospitalization or within 90 days of esophagectomy. Admission to the ICU during the postoperative period and readmission to the hospital within 30 days of discharge were recorded as well.

### Statistical analysis

Categorical variables were reported in absolute numbers and frequencies. Continuous variables were tested for presence of normal distribution, and reported as arithmetic means with standard deviation (SD), or median and interquartile range (IQR), as appropriate. Overall survival was calculated from the day of surgery to day of death, and censored at day of last follow-up for survivors. Disease-free survival was defined as the time from surgery to either disease recurrence or death, and censored at last follow-up. Overall and disease-free survival times were estimated by Kaplan Meier technique, and compared using log-rank test. The statistical analyses were performed using the SAS 9.2 statistical software package (SAS Institute, Cary, NC). Differences were considered significant when the probability was less than 0.05.

## Results

A total of 112 patients underwent total laparoscopic and thoracoscopic Ivor Lewis esophagectomy for locally advanced esophageal carcinoma after completing neoadjuvant chemoradiation. The patient demographics are listed in Table 1. The mean age of the patients was 61.6 years ± 10.4 years. The clinical staging based on endoscopic ultrasound and PET CT scan is listed in Table 1. Lymph Node involvement was based on abnormal radiotracer uptake on PET CT scans and endoscopic ultrasound findings of suspicious peri-esophageal lymphadenopathy. Fine needle aspiration was not attempted in most cases because the needle would have to be passed through large bulky tumors to reach the lymph nodes. The mean total operative time for patient who underwent total laparoscopic and thoracoscopic Ivor Lewis esophagectomy was 289.3 min ± 56.8 min. The median estimated blood loss was 50 mL. The mean number of lymph nodes dissected was 18.9 nodes ± 6.6. All of the patients had a R0 resection with negative proximal, distal, and radial margins on final pathology. Adenocarcinoma of was the most common histology comprising 93.75% of the cases (105/112).

**Table 1:** Patients Baseline Characteristics and Preoperative Data

Variable	Values (%)
Total No. of Patients (N)	112
Age, Mean (SD)	61.6 (10.4)
Age Group, N (%)	
< 60	42 (37.50)
60–69	47 (41.96)
> =70	23 (20.54)
Sex, N (%)	
Male	87 (77.68)
Female	25 (22.32)
BMI (Mean ± SD)	27.8 (5.9)
Smoking, N (%)	
Never	32 (28.57)
Former/Current	80 (71.43)
Diabetes, N (%)	
Yes	29 (25.89)
No	83 (74.11)
CAD, N (%)	
Yes	34 (30.36)
No	78 (69.64)
COPD, N (%)	
Yes	17 (15.18)
No	95 (84.82)
PET-CT, N (%)	
Yes	112 (100)
EUS, N (%)	
Yes	94 (83.93)
No	18 (16.07)
Histology, N (%)	
Adenocarcinoma	105 (93.75)
Squamous cell carcinoma	6 (5.36)
Neuroendocrine	1 (0.89)
Neoadjuvant CRT, N (%)	
Yes	112 (100)
Clinical Stage, N (%)	
I	3 (2.68)
II	12 (10.71)
III	97 (86.61)

Only 6.25% (7/112) of the patients were admitted to the ICU in the postoperative period. The overall operative 90–day mortality rate was 0.89% (1/112). A summary of the complications is summarized in Table 2. The overall complication rate was 25% (28/112). The total pulmonary complication rate was 1.79% (2/112). The anastomotic leak rate was 4/112 (3.57%). Two of the anastomotic leaks did not involve conduit necrosis and were successfully managed with esophageal stents. The other two cases with anastomotic leak did involve conduit necrosis which required a completion gastrectomy and esophageal diversion. One of the patients expired as a result of adult respiratory distress syndrome and sepsis. The other patient underwent a successful esophageal reconstruction with a jejunal interposition graft. There was one esophageal-airway fistula, which was managed with esophageal stent placement with complete resolution after 6 weeks. The readmission rate within 30 days after initial hospital discharge was 6.25% (7/112). None of the patients developed a chylothorax in the postoperative period. The complications were graded based on the Clavien-Dindo Severity Classification (Table 3). Most of the postoperative complications were Grade II requiring only pharmacological treatment. There was a Grade V complication or death that resulted from an anastomotic leak associated with conduit necrosis and sepsis.

**Table 2:** Perioperative Patient Characteristics

Variables, N (%)	Values
LOS, day (mean, SD), [median, IQR]	8.9 (3.5), 8.0 [7, 9]
Operating Time, Mean (SD), min	289.3 (56.8)
Ischemic Days, day (mean, SD), [median, IQR]	15 [14, 17]
Estimate Blood Loss, [median, IQR], ml	50 [50, 100]
Nodes Removed, Mean (SD)	18.9 (6.6)
Tumor Grade	
Well-differentiated	4 (3.57)
Moderately-differentiated	34 (30.36)
Poorly-differentiated	51 (45.54)
N/A (cannot be assessed)	23 (20.54)
Complications, N (%)	
Any Complications	28 (25.00)
Atrial fibrillation	19 (16.96)
Anastomotic Leak	4 (3.57)
Pneumothorax	2 (1.79)
Respiratory failure	1 (0.89)
Airway Fistula	1 (0.89)
Pneumonia	1 (0.77)
Atelectasis	0 (0.00)
Stricture	0 (0.00
ICU Admission, N (%)	
Yes	7 (6.25)
No	105 (93.75)
Readmission, N (%)	
Yes	8 (7.14)
No	104 (92.86)
Discharge within 24 h after LGD	
Yes	108 (96.43)
No	4 (3.57)
Margin	
Proximal positive margin	2 (1.79)
Distal positive margin	1 (0.89)
Lymphatic/vascular invasion	
Yes	31 (27.68)
No	81 (72.32)
Perineural invasion	
Yes	21 (18.75)
No	91 (81.25)
Recurrence Status	
Yes	21 (18.75)
No	91 (81.25)
Mortality with 90 days after surgery	
Died	1 (0.89)
Alive	111 (99.11)

*LGD* Laparoscopic gastric devascularization

**Table 3:** Clavien-Dindo Classification of Surgical Complications

Complication Severity	N	N %
No Complications	84	75
Grade I	0	0
Grade II	20	17.9
Grade IIIa	2	1.8
Grade IIIb	4	3.6
Grade Iva	1	0.89
Grade IVb	0	0
Grade V	1	0.89

Grade I: No need for pharmacological, surgical, or endoscopic treatment

Grade II: Requiring pharmacological treatment

Grade IIIa: Requires surgical, endoscopic, or radiological intervention without general anesthesia

Grade IIIb: Requires surgical, endoscopic, or radiological intervention with general anesthesia

Grade IVa: Single organ dysfunction

Grade IVb: Multi-organ dysfunction

Grade V: Death

**Fig. 1:**
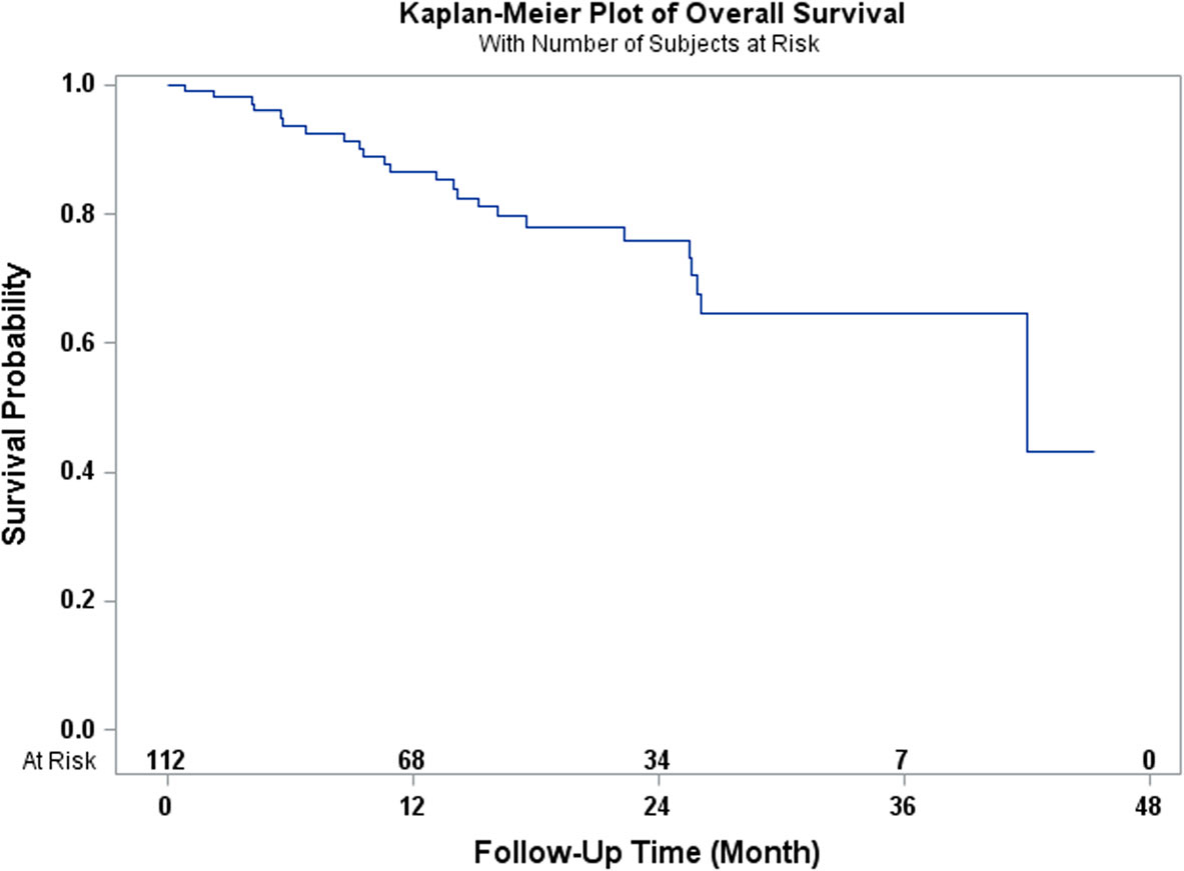
Kaplan-Meier Curve for Overall Survival

**Fig. 2:**
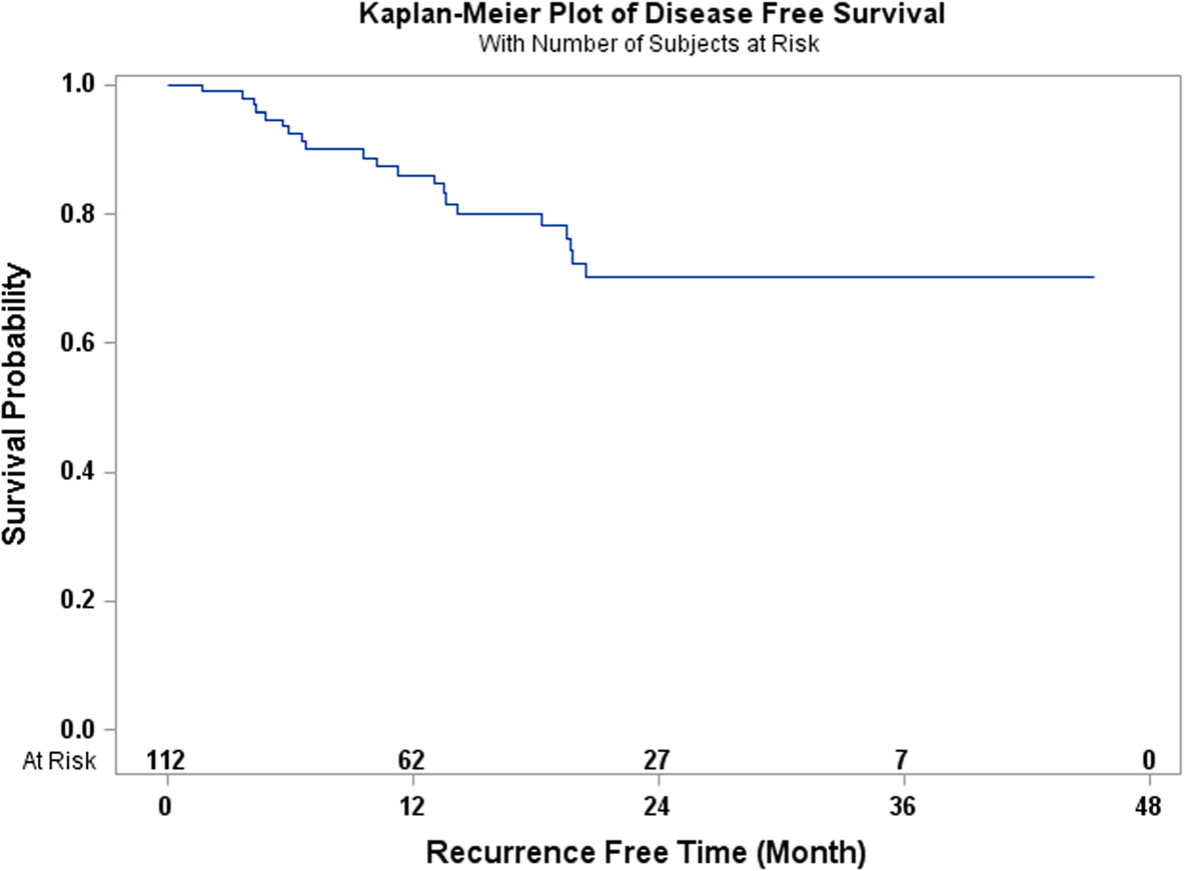
Kaplan-Meier Curve for Disease-Free Survival

**Fig. 3:**
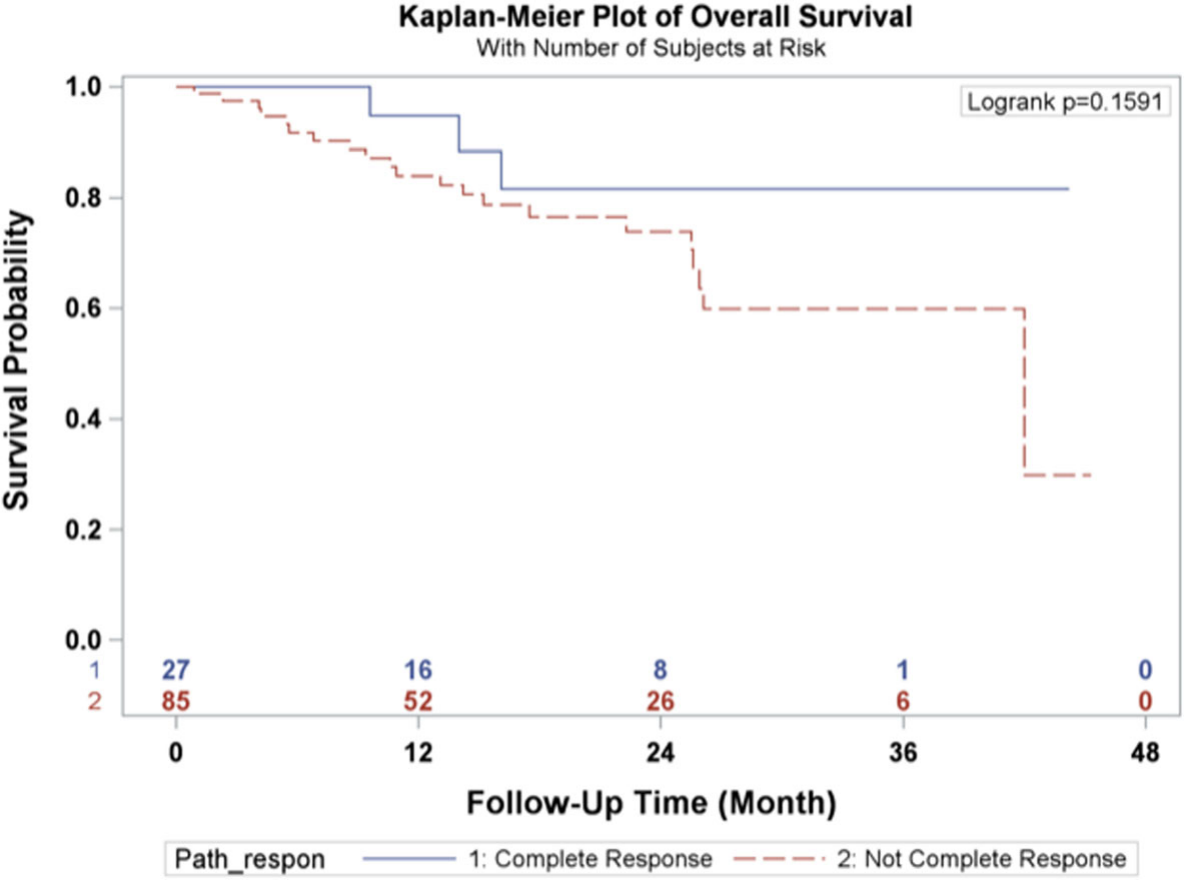
Pathologic Complete Response Association with Overall Survival

**Fig. 4:**
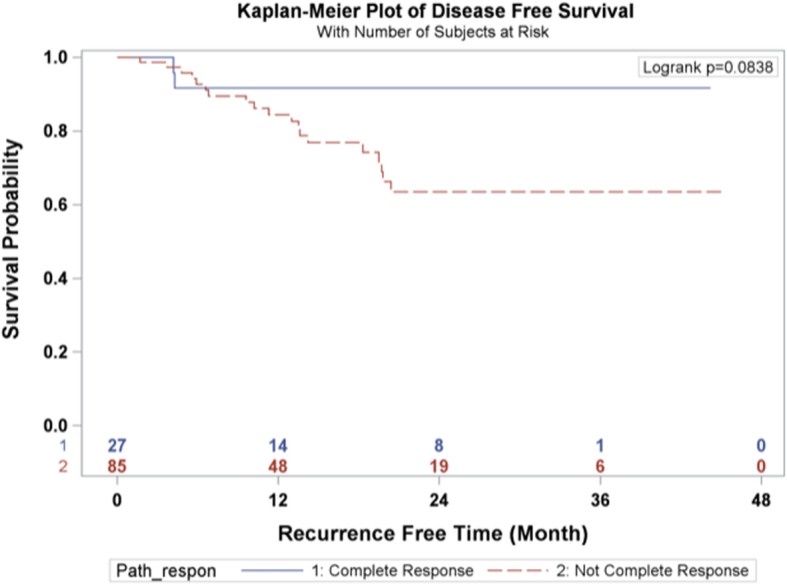
Pathologic Complete Response Association with Disease-Free Survival

**Fig. 5:**
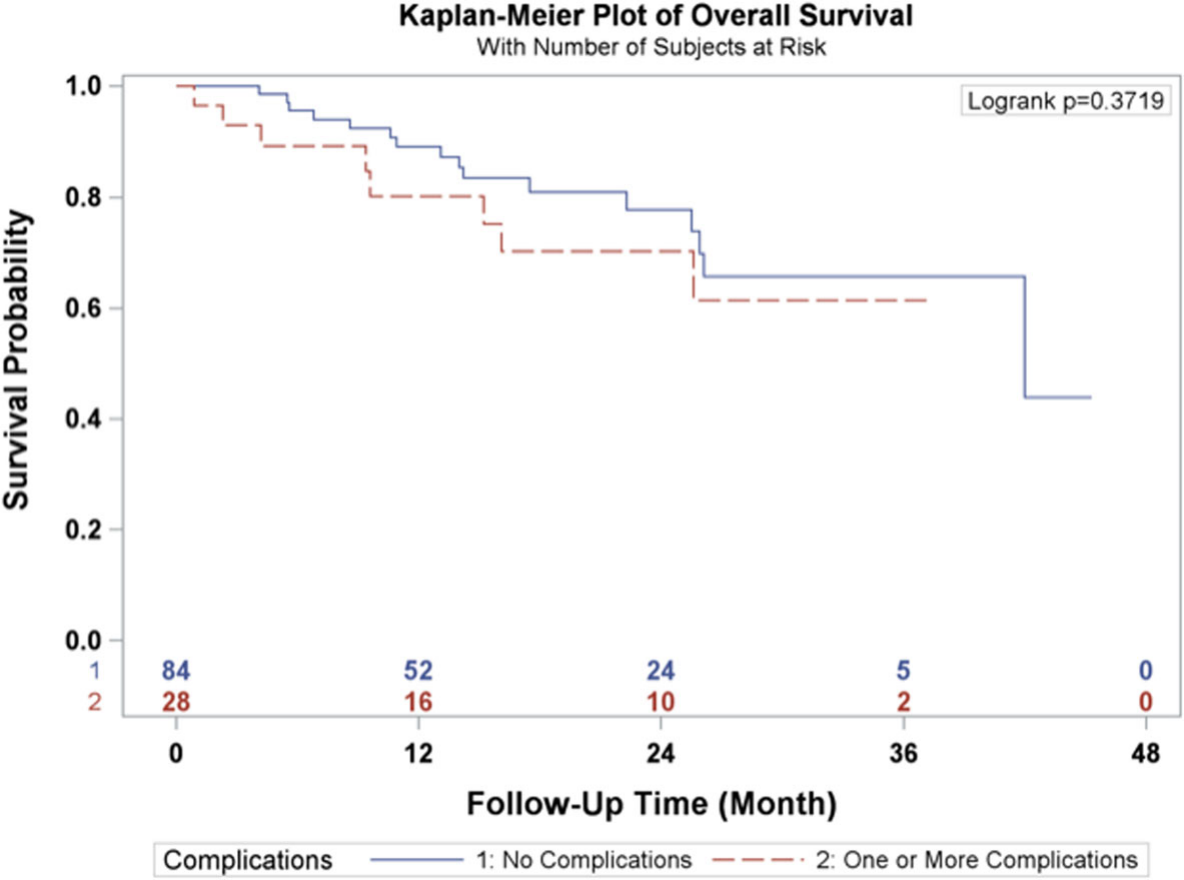
Complication Status Association with Overall Survival

**Fig. 6:**
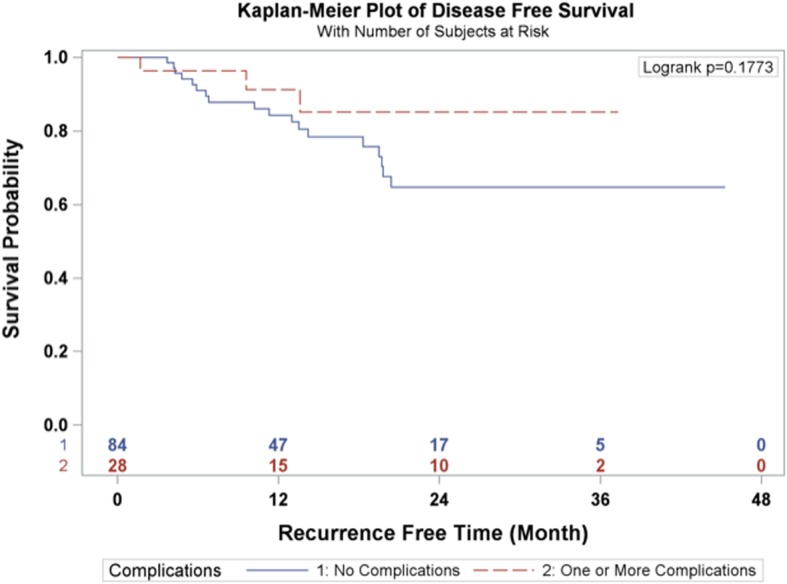
Complication Status Association with Disease-Free Survival

The 1-year and 3-year overall survival rates for esophageal carcinoma after total laparoscopic and thoracoscopic esophagectomy preceded by neoadjuvant chemoradiation was 86.6 and 64.7% respectively (Fig. 1). The 1-year and 3-year disease-free survival rates were 86.1 and 70.2% respectively (Fig. 2). A total of 27 patients (24.11%) had a complete pathologic response after neoadjuvant chemoradiation. The overall survival and disease-free survival rates were not significantly impacted by the pathologic response (Figs. 3 and 4). Similarly, the occurrence of postoperative complications did not significantly affect the overall survival and disease-free survival rates after neoadjuvant chemoradiation and minimally invasive Ivor Lewis esophagectomy (Figs. 5 and 6).

## Discussion

Since the first description of the two stage Ivor Lewis esophagectomy in 1946 [3], the procedure has become the procedure of choice at most centers for the resection of esophageal carcinoma involving the distal third of the esophagus and gastroesophageal junction. The main disadvantage of the open Ivor Lewis esophagectomy is the deleterious effects of the right thoracotomy. In a randomized clinical trial comparing transthoracic and transhiatal esophagectomy, Hulscher and colleagues demonstrated that patients undergoing esophagectomy with open thoracotomy had a significantly higher rate of pulmonary complications which resulted in more ventilator days, ICU days, and hospital days [2]. In an attempt to minimize the perioperative morbidity associated with esophagectomy, some centers have developed minimally invasive esophagectomy techniques for surgical resection of esophageal carcinoma. Luketich and colleagues reported their extensive experience with the total laparoscopic and thoracoscopic Ivor Lewis esophagectomy [5]. In this series, 530 patients with high-grade dysplasia or esophageal carcinoma underwent minimally invasive Ivor Lewis esophagectomy with relatively low operative mortality (0.9%). The median ICU days were 2 days and the median hospital length of stay was 7 days. These results for operative mortality rate and length of stay were superior to two large contemporary series involving open transthoracic and transhiatal esophagectomy [15, 16].

This report describes a large series of patients who underwent total laparoscopic and thoracoscopic Ivor Lewis esophagectomy for esophageal carcinoma after neoadjuvant chemotherapy with concurrent radiotherapy. The CROSS trial demonstrated an improved overall survival rate in patients with locally advanced esophageal carcinoma who received preoperative chemoradiation [2]. Despite the improved overall survival rate, there have been reports of increased incidence of anastomotic leak and increased pulmonary complication rates in patients who undergo esophagectomy after neoadjuvant chemoradiation [11, 12]. The authors wanted to determine if the complication rates and overall survival rates after laparoscopic and thoracoscopic Ivor Lewis esophagectomy were negatively impacted by neoadjuvant chemoradiation. In our report, 112 patients completed neoadjuvant chemoradiation, which consisted of multiple cycles of Carboplatin and Paclitaxel and concurrent 45–50.4 Gray of radiation. In our report, the overall 90-day mortality rate was 0.9%. A single patient died on postoperative day number 42 from Adult Respiratory Distress Syndrome and sepsis related to an anastomotic leak with associated gastric conduit necrosis. The overall complication rate was 25% and atrial fibrillation was the most common complication (17%). The intensive care unit admission rate was only 6.25% and the readmission rate was 7.1%. The overall pulmonary complication rate was 2.7%. The current published anastomotic leak rate ranged from 6.7 to 26% in other series reporting the outcomes in patients undergoing minimally invasive Ivor Lewis esophagectomy after neoadjuvant chemoradiation [17–20]. We observed only four postoperative anastomotic leaks (3.57%) in our series of 112 patients who underwent total laparoscopic and thoracoscopic Ivor Lewis esophagectomy after neoadjuvant chemoradiation. Two of the patients with anastomotic leaks required a completion gastrectomy and esophageal diversion due to associated conduit necrosis. The other two patients were managed successfully with esophageal stent placement without further intervention. The observed relatively low overall complication and anastomotic leak rates could potentially be related to the use of preoperative ischemic preconditioning and improved gastric conduit perfusion [13]. This technique may mitigate the deleterious effects of radiation therapy, which may negatively affect conduit perfusion and anastomotic healing.

The oncologic results of total laparoscopic and thoracoscopic Ivor Lewis esophagectomy were comparable to other published series. The mean numbers of lymph nodes that were dissected was 18.5 ± 6.6. All of the patients underwent a complete R0 resection with negative margins on final pathology. The Kaplan Meier overall survival at 3 years was 64.7% and the disease-free survival was 70.2% at 3 years. The randomized CROSS trial reported a 3 year overall survival rate of 58% for patients (*N* = 171) who underwent open esophagectomy after 5 cycles of carboplatin and paclitaxel and 41.4 Gy of radiation [2]. The overall complication rate was 46% and anastomotic leak rate was 22% in the preoperative chemoradiotherapy cohort. A direct comparison between our series would be difficult given the different study designs, patient selection, and operative techniques. The patients in the CROSS Trial underwent open thoracotomy which has been associated with increased pulmonary complications [4]. The overall pathological complete response rate in the CROSS trial was 29% compared to 24.11% in our series. The overall survival rates for patients who had a complete pathologic response after chemoradiation demonstrated improved overall survival and disease-free survival at 3 years compared to patients with residual esophageal carcinoma, which was not statistically significant (Figs. 3 and 4). This observation is most likely explained by the relative small sample size in our study. In addition, the presence of postoperative complications did not have any impact on the overall survival or disease-free survival at 3 years (Figs. 4 and 5). There was only one 90-day postoperative mortality and the overall rate of complications was relatively low in our series. Most of the postoperative complications were grade II based on the Clavien-Dindo Complication Severity Classification, which only required pharmacological therapy (Table 3).

## Conclusions

This report investigated the current perioperative and long-term outcomes of patients uniformly treated with neoadjuvant chemoradiation and total laparoscopic and thoracoscopic Ivor Lewis esophagectomy following gastric devascularization. The overall rate of complications and the anastomotic leak rate were lower relative to other published series. This retrospective study is unique because preoperative ischemic preconditioning was utilized to improve conduit perfusion and mitigate the risk of anastomotic leak. Regardless, the results of this report are limited by selection bias that is inherent to retrospective studies and a small sample size. This is a retrospective observational study; therefore, there is no comparison group. In a future study, we will compare the oncologic outcomes of minimally invasive Ivor Lewis esophagectomy to open Ivor Lewis esophagectomy performed at our institution. Based on the observed outcomes of this report, we conclude that total laparoscopic and thoracoscopic Ivor Lewis esophagectomy can be performed after neoadjuvant chemoradiation with minimal overall and anastomotic complications.

## Data Availability

The data used for this study is protected by United States federal patient privacy laws and cannot be shared publicly. The datasets used and analyzed during the current study are available from the corresponding author on a reasonable request.

## Abbreviations

CAD: Coronary Artery Disease
COPD: Chronic Obstructive Pulmonary Disease
DLCO: Diffusion Capacity
FEV1: Forced Expiratory Volume in One Second
ICU: Intensive Care Unit
PET: Positron Emission Tomography
